# Low loss photonic nanocavity via dark magnetic dipole resonant mode near metal

**DOI:** 10.1038/s41598-018-35291-w

**Published:** 2018-11-19

**Authors:** Ning Liu, Christophe Silien, Greg Sun, Brian Corbett

**Affiliations:** 1 Department of Physics and Bernal Institute, University of Limerick, Limerick, Ireland; 2 Department of Engineering, University of Massachusetts Boston, Boston, MA 02125 USA; 3 Tyndall National Institute, University College Cork, Cork, Ireland

## Abstract

The dielectric-semiconductor-dielectric-metal 4 layered structure is a well-established configuration to support TM hybrid plasmonic modes, which have demonstrated significant advantages over pure photonic modes in structures without metal to achieve low loss resonant cavities at sub-diffraction limited volumes. The photonic modes with TE characteristics supported by the same 4 layered structure, on the other hand, are less studied. Here we show that a low loss photonic mode with TE_01_ characteristics exists in the dielectric-semiconductor-dielectric-metal 4 layered structure if a truncated cylindrical disk is chosen as the semiconductor core. This mode exhibits the lowest cavity loss among all resonant modes, including both pure photonic and hybrid plasmonic modes, at cavity radius <150 nm and within the wavelength range 620 nm to 685 nm, with a footprint ~0.83 (*λ*/2n_eff_)^2^, physical size ~0.47 (*λ*/2n_eff_)^3^ and a mode volume down to 0.3 (*λ*/2n_eff_)^3^. The low cavity loss of this TE_01_ mode is attributed to its substantially reduced radiation loss to the far field by the creation of image charges through the metal response. Because of the low mode penetration in the metal, this photonic mode show equally low cavity loss near industry relevant metals such as Cu. Our study demonstrates an alternative to hybrid plamonic modes and metallo-dielectric modes to achieve low loss cavities with extremely small footprints.

## Introduction

Photonic lasers are one of the key components in integrated photonic circuits^1^. High integration densities of lasers on a chip will be needed for intra-chip communications. By increasing the refractive index of the lasing materials, the footprint of the photonic lasers can be decreased. However, due to the radiative nature of the dipole mode, pure photonic lasers cannot maintain an effective feedback as the dimension of the cavity gets close to (*λ*/2*n*_*eff*_)^3 ^^2^. As a result, conventional optical cavities can hardly achieve lasing at dimensions around (*λ*/2*n*_*eff*_)^3^. To overcome this limitation, metal coated photonic cavities, termed metallo-dielectric cavities, have been exploited to confine light into a diffraction limited volume of (*λ*/2*n*_*eff*_)^3 ^^3–7^.

Alternatively, people have been looking into hybrid plasmonic modes, which exist at the dielectric-semiconductor-dielectric-metal interface, to achieve lasing at deep subwavelength scale with low propagation loss, promising substantial increase in packing density of optical components in integrated circuits. Since the first experimental observation of hybrid plasmonic lasers by Oulton *et al*.^8^, progress has been made to demonstrate greater mode confinement down to 0.2(*λ*/2*n*_*eff*_)^3^ in the wavelength range of 370 nm to 685 nm and of 1340 nm in the Infrared^9–15^. Nevertheless, the extreme mode confinement offered by hybrid plasmonic modes is mostly achieved in 1D or 2D. As a result, the footprint of this type of laser is usually larger than (*λ*/2*n*_*eff*_)^2^. Different from the well-studied hybrid plasmonic mode, which has mostly transverse magnetic (TM) characteristics, the photonic mode that co-exists in this type of structure, which has transverse electric (TE) characteristics, on the other hand, has received little attention, other than being used as the comparison to the hybrid plasmonic mode to show that the effective refractive index in the photonic case is lower than that in the hybrid plasmonic mode^12^.

In this report, we focus our attention on the photonic mode that exists in the dielectric-semiconductor-dielectric-metal 4-layered structure and demonstrate how this photonic mode can be used to achieve low loss, sub-diffraction nanocavities with very small footprints.

## Results

### Analytical model of Fundamental TE mode in a 4-layered planar structure

**Figure 1:**
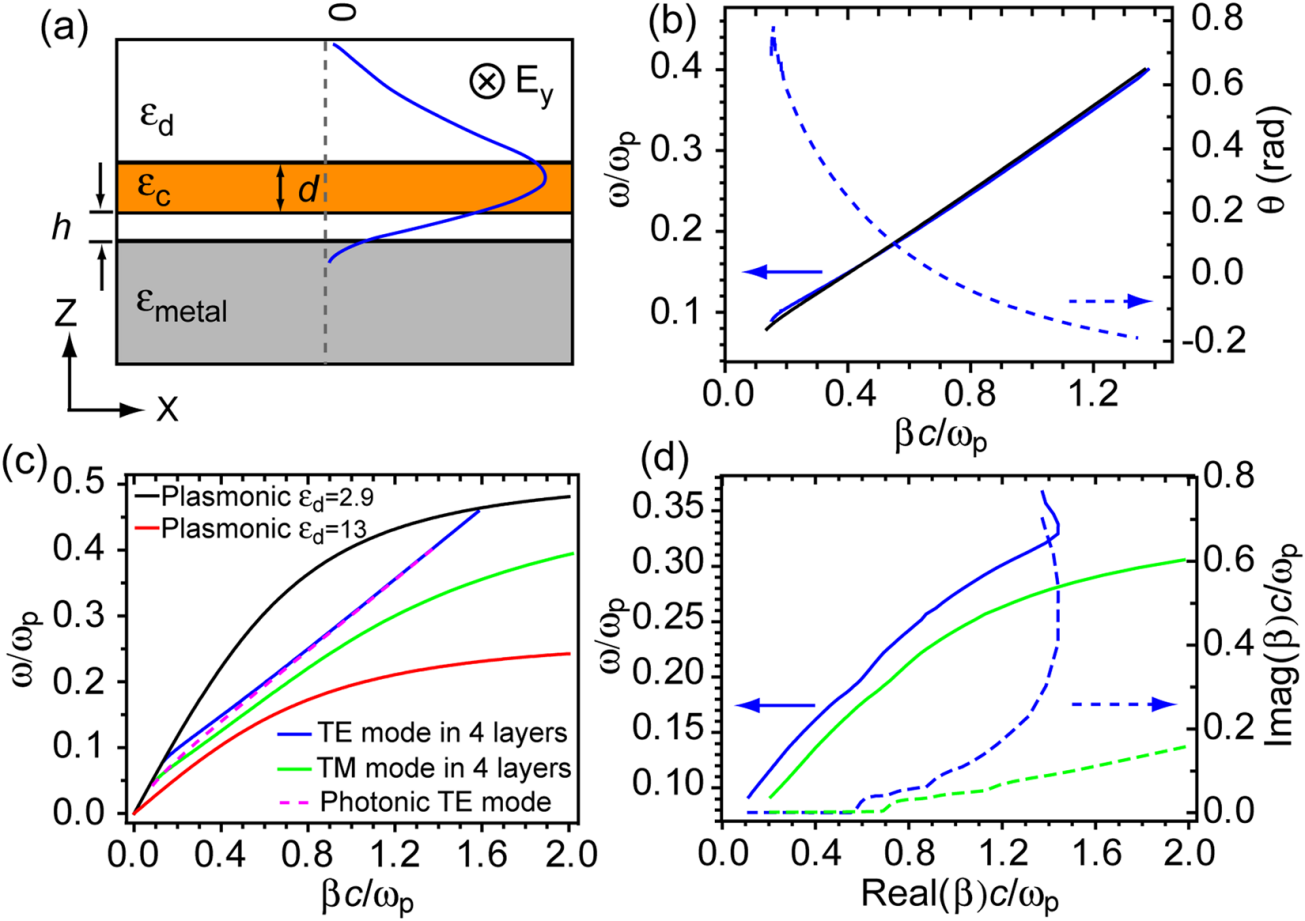
TE mode in the dielectric-semiconductor-dielectric-metal 4 layered planar geometry and its dispersion relationship. (**a**) Diagram of the TE mode electric field distribution within the dielectric-semiconductor-dielectric-metal 4 layered structure. (**b**). Dispersion curves of TE mode from Eq. (7) (blue, angle *θ* shown on the right axis) and from COMSOL simulation (black). (**c**) Dispersion curves of plasmonic modes at single dielectric-metal interface with permittivity ε_d_ = 2.9 (black) and ε_d_ = 13 (red), TE mode in the 4 layered structure (blue), TM mode in the 4 layered structure (green) and photonic TE mode in a dielectric-semiconductor-dielectric 3 layered structure (dashed pink). In simulations for (**b** and **c**), we ignore the loss of the metal and use ε_m_ = 1 − ω_p_^2^/ω^2^ with ω_p_  = 1.4 × 10^16^ rad·Hz. For 3 layered structure, we use ε_d_ = 2.9, ε_c_ = 13, *d* = 110 nm and an additional *h* = 6 nm for 4 layered structure. (**d**) Dispersion curves of TE (blue) and TM (green) modes in an Air-GaInP-Al_2_O_3_-Ag 4 layered structure. The permittivities of GaInP^18^ and Ag^8^ are from realistic material parameters. The right axis shows the losses of the modes (dashed curves).

To understand this photonic mode, we first seek solutions to the Helmholtz equation ${\nabla }^{2}{\bf{E}}+\frac{{\omega }^{2}\varepsilon }{{c}^{2}}{\bf{E}}=0$ in a 4 layered planar structure of dielectric-semiconductor-dielectric-metal, as shown in Fig. 1a. We define that the light propagates in x direction only and there is no spatial variation in the y direction. The z axis is perpendicular to the interface of different media. In this configuration, both TE and TM optical modes exist. The TM mode in this structure can be considered as the hybridization of the guided photonic TM mode in the top three layers with the TM plasmonic mode at the interface of the bottom two layers. This TM mode is commonly called a hybrid plasmonic mode as its dispersion curve closely resembles that of the surface plasmonic propagation at a single dielectric-metal interface^16^ (also shown later in Fig. 1c).

To obtain the TE mode solution, we solve the Helmholtz equation in these 4 different regions separately and require the solution to satisfy the continuity of E_*y*_ and H_*x*_ at the interfaces. To simplify our calculations, we choose the refractive index of the dielectrics to be the same *n*_dielectric1_ = *n*_dielectric2_ = *n*_d_. It is well known that the single dielectric-metal interface does not support a TE mode^17^. In the 4 layered structure with a higher refractive index of semiconductor core layer, however, the continuity boundary conditions at the dielectric-metal interface can be satisfied. If we choose z = 0 at the middle of the semiconductor layer and let d = 2a (Fig. 1a), the solution of the fundamental TE mode in this planar structure can be written as (see Supplementary Information 1 for the Hz components):

$$\{\begin{array}{ll}{E}_{y}(z)={B}_{t}{e}^{i\beta x}{e}^{-{k}_{d}(z-a)}\, & {\rm{for}}\,z > a\\ {E}_{y}(z)=A{e}^{i\beta x}\,\cos ({k}_{c}z-\theta )\, & {\rm{for}}\,|z| < a\\ {E}_{y}(z)={B}_{b1}{e}^{i\beta x}{e}^{{k}_{d}(z+a)}+{B}_{b2}{e}^{i\beta x}{e}^{-{k}_{d}(z+a)}\, & {\rm{for}}\,-\,(a+h) < z < -\,a\\ {E}_{y}(z)=C{e}^{i\beta x}{e}^{{k}_{m}(z+a+h)}\, & {\rm{for}}\,z < -\,(a+h)\end{array}\,$$

$$\{\begin{array}{ll}{H}_{x}(z)=-\,i{B}_{t}\frac{{k}_{d}}{\omega {\mu }_{0}}{e}^{i\beta x}{e}^{-{k}_{d}(z-a)}\, & {\rm{for}}\,z > a\\ {H}_{x}(z)=-\,iA\frac{{k}_{c}}{\omega {\mu }_{0}}{e}^{i\beta x}\,\sin ({k}_{c}z-\theta )\, & \mathrm{for}\,\,|z| < a\\ {H}_{x}(z)=i{B}_{b1}\frac{{k}_{d}}{\omega {\mu }_{0}}{e}^{i\beta x}{e}^{{k}_{d}(z+a)}-i{B}_{b2}\frac{{k}_{d}}{\omega {\mu }_{0}}{e}^{i\beta x}{e}^{-{k}_{d}(z+a)} & {\rm{for}}\,-\,(a+h) < z < -\,a\\ {H}_{x}(z)=iC\frac{{k}_{m}}{\omega {\mu }_{0}}{e}^{i\beta x}{e}^{{k}_{m}(z+a+h)}\, & {\rm{for}}\,z < -\,(a+h)\end{array}$$

Here ${k}_{i}^{2}={\beta }^{2}-\frac{{\omega }^{2}{\varepsilon }_{i}}{{c}^{2}}$ (*i* = *d*, *m*) and ${k}_{c}^{2}=\frac{{\omega }^{2}{\varepsilon }_{c}}{{c}^{2}}-\,{\beta }^{2}$. *β* is the propagation constant and ${\varepsilon }_{j}$ (*j* = *d*, *c*, *m*) is the relative permittivity of the dielectric, semiconductor core and metallic substrate, respectively. *B*_*t*_, *A*, *B*_*b1*_, *B*_*b2*_ and *C* are coefficients related to the amplitude of the mode in four regions and *θ* is a parameter describing how far away the maximum of electric field is off the center of the semiconductor core layer due to the presence of bottom metallic layer. The continuity of *E*_*y*_ and *H*_*x*_ at three interfaces leads to:

1 $${B}_{t}=Acos({k}_{c}a-\theta )\,$$

2 $${B}_{b1}+{B}_{b2}=Acos({k}_{c}a+\theta )\,$$

3 $${B}_{b1}{e}^{-{k}_{d}h}+{B}_{b2}{e}^{{k}_{d}h}=C\,$$

and

4 $${B}_{t}{k}_{d}=A{k}_{c}sin({k}_{c}a-\theta )\,$$

5 $${k}_{d}({B}_{b1}-{B}_{b2})=A{k}_{c}sin({k}_{c}a+\theta )\,$$

6 $${k}_{d}({B}_{b1}{e}^{-{k}_{d}h}-{B}_{b2}{e}^{{k}_{d}h})=C{k}_{m}\,$$

These 6 equations result in the dispersion relationship of this TE mode:

7 $$\frac{2{k}_{c}+tan({k}_{c}a){k}_{d}-ctan({k}_{c}a){k}_{d}}{2{k}_{d}-tan({k}_{c}a){k}_{c}+ctan({k}_{c}a){k}_{c}}=\,\frac{{k}_{d}+{k}_{m}-({k}_{d}-{k}_{m}){e}^{-2{k}_{d}h}}{{k}_{d}+{k}_{m}+({k}_{d}-{k}_{m}){e}^{-2{k}_{d}h}}$$

The solution to Eq. (7) can be obtained numerically via the Newton-Raphson method. As a proof of concept, we calculate the dispersion curve of this TE mode from Eq. (7) with a chosen set of parameters and compare the results with those directly obtained from COMSOL multiphysics simulation package (see Methods for details), as shown in Fig. 1b. To simplify the numerical calculation from Eq. (7), all dielectric materials and metal are treated lossless in Fig. 1b. The dispersion curves obtained from these two methods agree very well, with a discrepancy of less than 5% in all frequencies shown in the plot. The obtained angle *θ* is also plotted in the same panel as a function of *β*. From the plot, we can also see that at lower frequencies (longer wavelengths), *θ* > 0 and the center of the photonic mode is pushed slightly away from the center of the semiconductor core layer and in the direction away from the metal substrate. This leads to a decrease in the effective refractive index of the TE mode in the 4-layered structure compared to that of a typical guided photonic TE mode in dielectric-semiconductor-dielectric 3-layered structure.

As a comparison to other optical modes, a set of simulated dispersion curves of the 3-layered photonic TE mode, 4-layered TE mode and 4-layered TM mode (hybrid plasmonic mode) are shown in Fig. 1c (see Supplementary Information 2,3 for details). It is clear from Fig. 1c that the 4-layered TE mode gives the lowest effective refractive index among these three at a given frequency especially in the low frequency region, consistent with the results previously reported on this TE mode^12^.

For real materials, ${\varepsilon }_{j}$ (*j* = *d*, *c*, *m*) are all complex numbers, resulting in *β* complex in the solution to Eq. (7). To evaluate the influence of the losses in materials to the dispersion relationship of the TE mode, we simulate the dispersion curve in this 4-layered TE mode using realistic material parameters of GaInP, from ref.^18^ as the semiconductor core layer, Ag (${\varepsilon }_{Ag}={\varepsilon }_{\infty }-\,\frac{{\omega }_{p}^{2}}{{\omega }^{2}+i\gamma \omega }$, where ${\varepsilon }_{\infty }=5\,\,$and γ = 0.04 eV)^8^, Al_2_O_3_ ultrathin layer of 6 nm (${\varepsilon }_{A{l}_{2}{O}_{3}}=2.9$) as the dielectric layer between GaInP and Ag, and Air (${\varepsilon }_{Air}=1$) as the top dielectric layer. As a comparison, the TM mode supported by the same 4 layered planar structure is also simulated and plotted together in Fig. 1d. Figure 1d shows that the dispersion curves of TE and TM modes follow a very similar trend as those in Fig. 1c (without losses) in general, but are modulated by the change in refractive index of GaInP at higher frequencies. The imaginary part of the propagation constant *β* indicates the overall losses of the modes, which is due to the absorption of GaInP and ohmic loss of the metal.

### TE_01_ and TM_11_ modes in a trancated cylindrical cavity near metal

**Figure 2:**
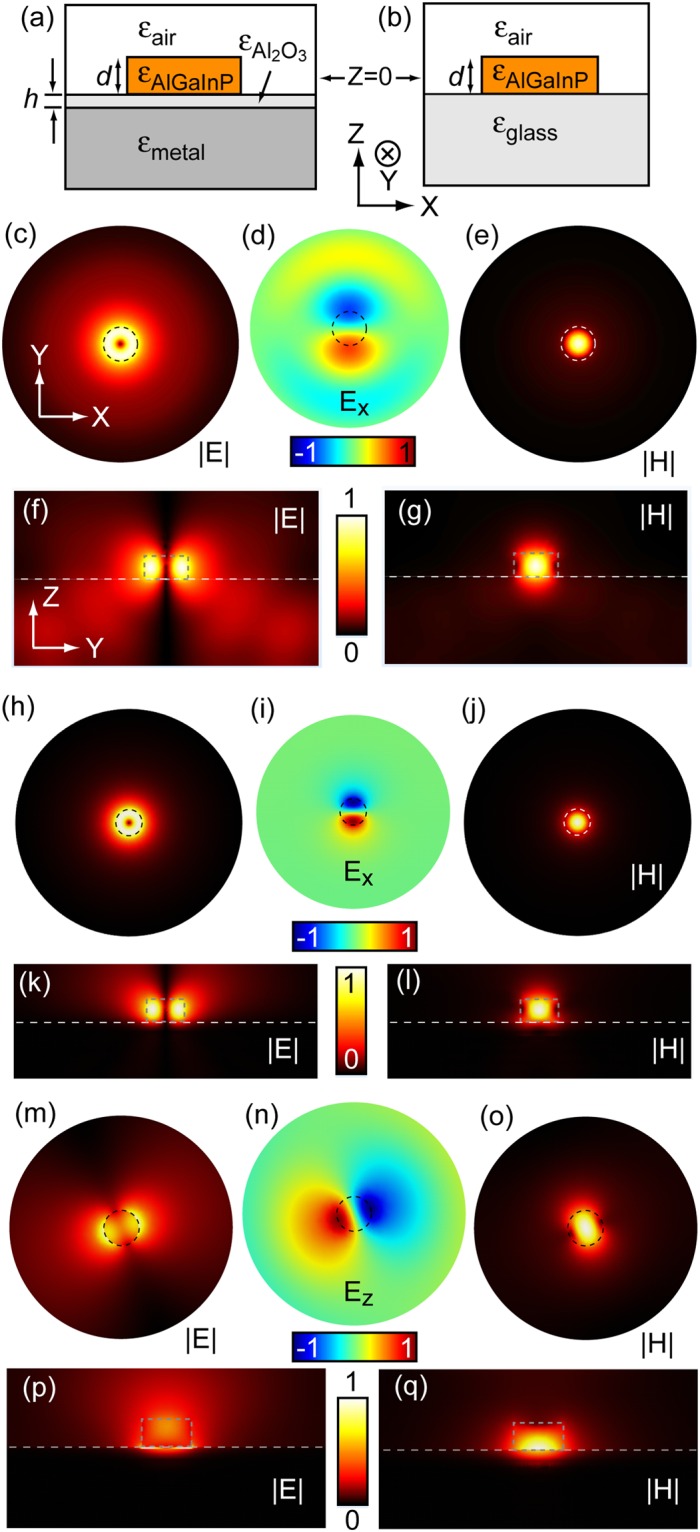
Electric and magnetic field distribution of TE_01_ mode supported by semiconductor disk on glass, Ag and TM_11_ mode on Ag. Diagrams of a truncated cylindrical semiconductor disk near metal (**a**) and on glass (**b**). z = 0 is chosen at the middle of the semiconductor disk. (**c**–**e**) Pure photonic TE_01_ electric field amplitude |E|, electric field x component E_x_ and magnetic field amplitude |H| distribution on xy plane at z of 45 nm into the glass, respectively. (**f,g**) TE_01_ electric field amplitude |E| and magnetic field amplitude |H| distribution on zy plane at x = 0 nm, respectively. (**h**–**j**) Near metal TE_01_ electric and magnetic field distribution on xy plane at z = −15 nm, respectively. (**k,l**) TE_01_ electric and magnetic field amplitude distribution on zy plane at x = 0 nm, respectively. (**m**–**o**) Near metal TM_11_ electric field amplitude, electric field z component E_z_ and magnetic field amplitude distribution on xy plane at z = −15 nm, respectively. (**p,q**) TM_11_ electric and magnetic field amplitude distribution on zy plane at x = 0 nm, respectively. The electric field peaks within the Al_2_O_3_ layer, showing the characteristics of a hybrid plasmonic mode. The physical dimension of the semiconductor disk is highlighted by dashed curves in (**c**–**q**). The white dashed lines indicate the interface between the semiconductor disk and the substrate. In all simulations, the diameter of the disk is 200 nm. To facilitate the direct comparison with previous work^13^, we keep the Al_2_O_3_ thickness fixed at 6 nm.

Interesting results appear when we use the 4-layered structure to form nanocavities. In the following simulations, we keep the metal substrate (Ag) optically thick and laterally large and shrink the lateral dimension of the semiconductor layer to form a truncated cylindrical disk shaped cavity (Fig. 2a). In this design, the top dielectric layer is air and the bottom dielectric layer is Al_2_O_3_ of a thickness of 6 nm, same as the values used in Fig. 1d. The thickness of the semiconductor layer is kept at 110 nm and the semiconductor material is chosen to be the quaternary AlGaInP as used in ref.^13^. As a comparison, we also investigate pure photonic cavities of same semiconductor core dimensions, as shown in Fig. 2b. Similar to Fig. 1d, simulations below use realistic material parameters, as used in refs^8,13^. If we look for resonant modes in a fixed range of wavelengths, for example 620 nm to 685 nm for the active region of quaternary AlGaInP, we found previously that for cavities of diameter larger than 1 µm, the Q of the pure photonic cavities is much higher than that of the cavities near metal. As the diameter of the cavity decreases below the wavelength of the corresponding cavity mode, the cavity loss in the photonic cavity increases rapidly due to the loss of mode confinement and more importantly the increase of radiation loss^19^. On the other hand, because of the higher effective refractive index offered by the hybrid plasmonic modes, the higher order (≥3) whisper gallery resonant modes of hybrid plasmonic characteristics are the least lossy for cavities of diameter between 300 nm and 650 nm^13^. For cavities below the diameter of 300 nm, the whispering gallery resonant mode number drops below 3. As a result, the mode confinement starts to deteriorate (effective refractive index n_eff_ drops below 1.7) and total internal reflection is no longer satisfied. Consequently, the quality factor Q of the hybrid plasmonic resonant modes decreases rapidly^13^.

At very small cavity diameters, namely from 190 nm to 240 nm, a low loss resonant TE mode in the 4 layered structure starts to emerge in the wavelength range of 620 nm to 685 nm. Solving the Helmholtz equation in cylindrical coordinates (*ρ*, *φ*, z) reveals that this TE resonant mode can be described by the fundamental TE_01_ mode, which is also called a magnetic dipole mode^5,6^. (see Methods for details) Its H_z_ component within the semiconductor core can be approximated by a Bessel function ~J_0_(*k*_ρc_*ρ*) and E_φ_ component ~J_1_(*k*_ρc_*ρ*), while the H_z_ component outside the semiconductor core can be mostly approximated by the modified Bessel function of second kind ~K_0_(*k*_ρd_*ρ*) and E_φ_ component ~K_1_(*k*_ρd_*ρ*). Here *k*_ρc_ and *k*_ρd_ are determined by permittivities of the semiconductor core and outer dielectric material. (see Methods for details) This TE_01_ mode distribution mimics a classical circular electric current (the donut shape in Fig. 2c) inducing a vertical magnetic field (Fig. 2g).

Without the metal substrate, this TE_01_ mode is of radiative nature (Fig. 2c–g). As the length scale of the cavity approaches λ/2*n*_*eff*_, the cavity becomes a good antenna. If we consider the cavity losses as the sum of internal losses (due to the absorption of semiconductor disk) and radiation loss^20^, the latter dominates for the semiconductor disk with diameter >200 nm. Figure 2d shows the E_x_ distribution on xy plane with z at 45 nm into glass substrate for a semiconductor disk of 200 nm in diameter on glass. The radiation pattern from the disk is clearly visible from the plot. The leak of energy into the glass substrate is also observable in Fig. 2f. This radiation loss places a fundamental limit to the quality factor of the TE_01_ mode in a pure photonic cavity.

**Figure 3:**
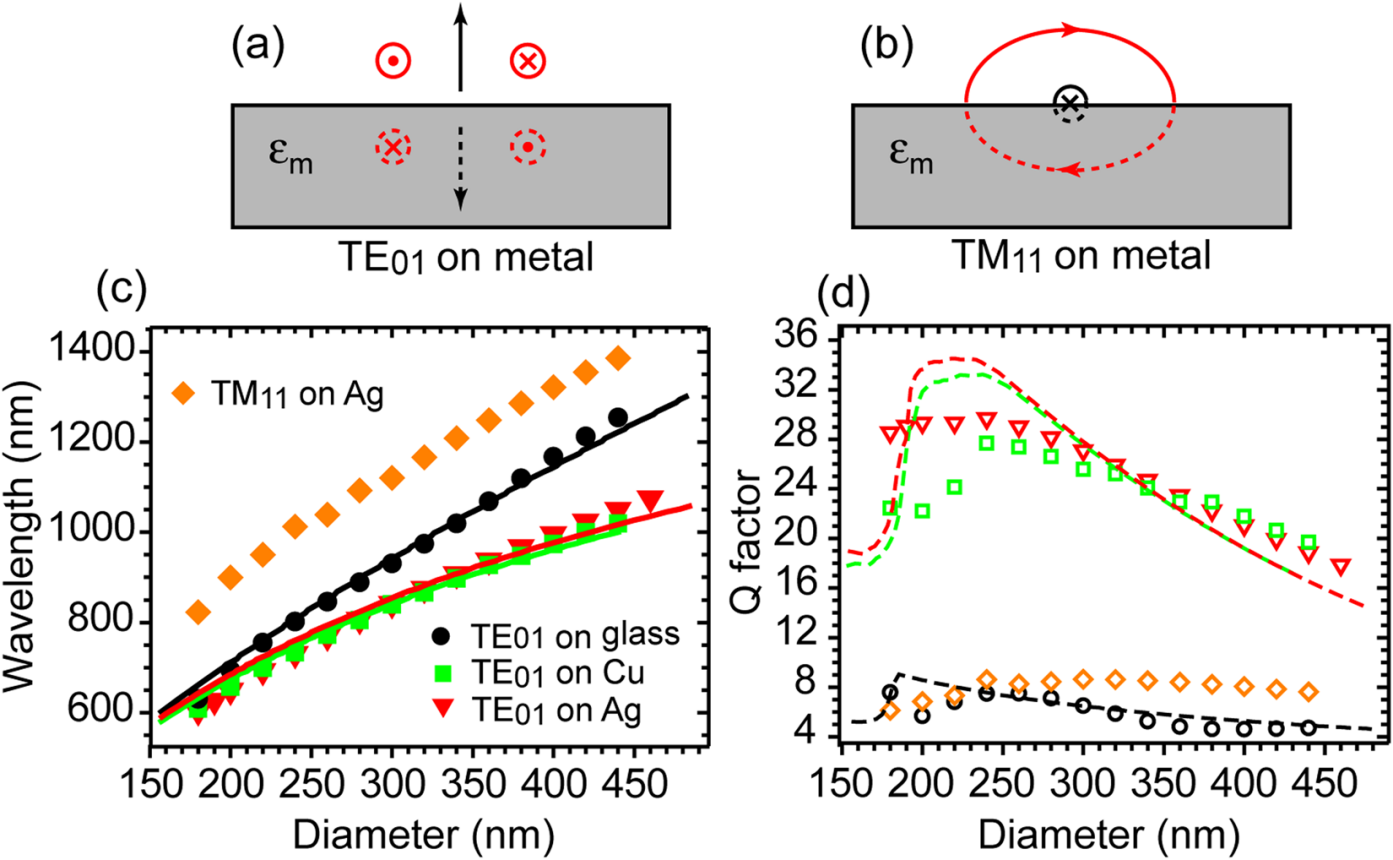
Comparison of resonant wavelength and quality factor of TE_01_ mode on glass, metal and TM_11_ mode on Ag. (**a**,**b**) Diagrams showing the image charges creating an opposing or parallel electric field, and therefore a magnetic dipole in either opposite (**a)** or same direction (**b**). The red symbols indicate the electric field and black symbols the magnetic dipole direction. The dashed circles and arrow correspond to the electric field and magnetic dipole created by the image charges. (**c**) Simulated (using COMSOL) resonant wavelength (solid symbols) and (**d**) corresponding quality factors Q (hollow symbols) of TE_01_ mode supported by the semiconductor nanodisk on glass (black circle), on Al_2_O_3_/Ag (red triangle), on Al_2_O_3_/Cu (green square) and those of TM_11_ mode supported by the semiconductor nanodisk on Al_2_O_3_/Ag (orange diamond) as a function of disk diameters, respectively. The fittings to the resonant wavelength of TE_01_ mode on glass, on Al_2_O_3_/Ag and on Al_2_O_3_/Cu and those to their corresponding quality factor Q as a function of disk diameter, following analytical models detailed in Methods, are presented as solid (**c**) and dashed (**d**) curves, respectively.

With the semiconductor disk placed on top of a metal substrate, the metal responds to electromagnetic oscillations in the cavity by generating image charges at the interface. The image charges create an opposing electric field in the cavity. An opposite magnetic field, as illustrated in Fig. 3a, is also generated. Consequently, the radiation of this magnetic dipole mode to the far field is strongly suppressed due to its image magnetic dipole (in opposite direction). This is evident in Fig. 2h,i, where the electric field shows little distribution outside the semiconductor core. We therefore call this TE_01_ mode a ‘dark’ magnetic dipole mode. We also calculate the footprint, physical size and effective mode volume^13^ of the TE_01_ mode as shown in Fig. 2h–l, which are 0.83(*λ*/2n_eff_)^2^, 0.47(*λ*/2n_eff_)^3^ and 0.3(*λ*/2n_eff_)^3^ respectively. The TM_11_ resonant mode in this structure, on the other hand, shows much higher cavity loss due to the enhanced radiation, as illustrated in Fig. 3b, where the vertical component of electric field induces an imaging electric field of the same direction. As a result, an effective lying down magnetic dipole is induced. This mode is similar to the electric dipole induced magnetic dipole mode observed on silicon nanocavities when placed on top of a metallic mirror^21,22^. The enhanced radiation loss is also evident in the field distribution plots of Fig. 2m–q. The electric field spills out of the physical dimension of the semiconductor core (Fig. 2m) and radiates out to the far field (Fig. 2p).

## Discussion

Figure 3 compares the simulated resonant wavelengths (Fig. 3c) of the TE_01_ mode on glass, on Al_2_O_3_/Ag, on Al_2_O_3_/Cu and the TM_11_ mode on Al_2_O_3_/Ag as well as their corresponding quality factors Q (Fig. 3d) as a function of the disk diameter. It is clear from the plots that the TE_01_ mode supported by disks on glass shows the highest effective refractive index compared to that of the TE_01_ modes supported by disks on Al_2_O_3_/Ag or Al_2_O_3_/Cu, at a given diameter, consistent with the conclusion we obtain from the planar structures in Fig. 1. Nevertheless, the quality factors Q of the TE_01_ modes near metal are substantially higher compared to that of its counterpart on glass. Notably, because this TE_01_ ‘dark’ magnetic dipole mode has photonic characteristics and the mode overlap with the metal is very small, the requirement for ‘good’ plasmonic metals, such as Ag and Au, to maintain its low cavity loss and mode confinement is relaxed. As shown in Fig. 3d, the quality factor Q of TE_01_ mode on industry relevant metal Cu^23^ is comparable with that on Ag. Analytical models to estimate the resonant wavelength of TE_01_ mode on glass, that on Al_2_O_3_/Ag and on Al_2_O_3_/Cu as well as their corresponding quality factor Q, taking into account both the radiation loss of the magnetic dipole and internal losses of metal and semiconductor, as a function of disk diameter are proposed and detailed in the Methods. Fittings of simulated TE_01_ resonant wavelengths to the analytical models are given in Fig. 3c as solid curves. Plugging the fitted parameters to the analytical loss model yields the quality factor Q of TE_01_ modes on various substrates, indicated by the dashed curves in Fig. 3d. It is understood from the analytical models that for semiconductor disks of diameter ≥250 nm, the radiation loss dominates in the TE_01_ cavity loss while for diameter <200 nm, the internal losses of the cavity prevail. The simulated Q by COMSOL package shows a similar trend. We also find that increasing the Al_2_O_3_ spacer layer thickness slightly decreases the Q of the TE_01_ mode, which can be understood as the increase of distance between the magnetic dipole and its image dipole leading to inefficient suppression of the radiation loss to the far field. (see Supplementary Figure S3).

The TM_11_ mode on Al_2_O_3_/Ag, on the other hand, demonstrates strong hybridization between the photonic mode and surface plasmonic mode, resulting in much higher effective refractive index compared to that of TE_01_ mode on glass or near metal (Fig. 3c). However, the enhanced radiation loss in the TM_11_ mode near metal also results in higher cavity loss therefore much lower cavity quality factor Q in the full diameter range of Fig. 3d compared to that of TE_01_ mode on Al_2_O_3_/Ag. Consequently, the TE_01_ ‘dark’ magnetic dipole mode becomes more advantageous over the hybrid plasmonic TM_11_ mode or the photonic TE_01_ magnetic dipole mode as the diameter of the cavity decreases below 250 nm.

The lowest quality factor Q of cavities that achieved lasing at room temperature using similar materials as discussed here is ∼79, with an oval-shaped 350 nm × 390 nm quaternary AlGaInP disk on Al_2_O_3_/Ag^13^. The simulated Q of TE_01_ modes (within the wavelength range of 620 nm to 685 nm) on Ag and Cu varies from 24 to 29, which is difficult to achieve lasing at room temperature. Nevertheless, Lu *et al*. have reported hybrid plasmonic lasers achieved at 120 K with cavity Q-factor of ∼13 in the wavelength range of 470 nm to 640 nm^10,11^. It is therefore reasonable to assume that at low temperature, for example 120 K, where the resistive heating in metal is greatly reduced^6^, and with better metal preparation^10^, lasing from the TE_01_ ‘dark’ magnetic dipole mode can be expected. It needs to be noted that the technical advantage of the 4-layered structure lies in the fewer processing steps compared to the steps required for the top and bottom capped metal-clad structures. If the extreme footprint is the ultimate goal of the cavity design, the metal-clad metal–insulator–semiconductor mode can be a better option^6,24,25^.

In conclusion, we theoretically demonstrate that low loss photonic modes exist in dielectric material of finite dimensions near metal. Using truncated cylindrical disk geometry, we can form photonic nanocavities of TE_01_ characteristics with extremely small footprint of 0.83(*λ*/2*n*_*eff*_)^2^, a physical size of 0.47(*λ*/2*n*_*eff*_)^3^ and subwavelength mode volume of 0.3(*λ*/2*n*_*eff*_)^3^ while maintaining low cavity losses through the creation of image charges in metal. In contrast to the case when a magnetic dipole mode is aligned in parallel with the metallic substrate its scattering cross section is strongly enhanced^22^; when the magnetic dipole mode is perpendicular to the metallic substrate, its image dipole is opposite to the original one and the radiation loss of the cavity to the far field is substantially suppressed. This type of photonic mode is analogous to the metallo-dielectric cavity in nature. Without the top metal coating and insulating layers, however, the fabrication steps can be considerably reduced. Our findings demonstrate a promising alternative to metallo-dielectric modes or hybrid plasmonic modes for the realization of nano-lasers using industry relevant materials and techniques.

## Methods

### COMSOL simulation

Wave optics module in COMSOL Multiphysics 5.2 simulation package is used for finite element simulations. To calculate the dispersion curves in the planar structures, effective mode indices in 2D simulation are obtained at different frequencies. The resonant frequencies, mode losses and mode distributions at various cavity diameters are obtained via 3D eigenfrequency calculation. In simulations for Fig. 1d and 3D simulations in Fig. 2 and Fig. 3, the permittivities of GaInP, AlGaInP, Ag and Cu are obtained via realistic material parameters as described in ref.^18^, ref.^13^, ref.^8^ and ref.^23^, respectively.

### Guided TE photonic mode in a cylindrically shaped dielectric waveguide

To solve the Helmholtz equation in cylindrical coordinates (*ρ*, *φ*, z) with z direction as the propagation direction, we first need to solve for H_z_ component for a TE mode, which satisfies inside and outside the core^19,26^:

$$\frac{{\partial }^{2}{H}_{z}}{\partial {\rho }^{2}}+\frac{1}{\rho }\frac{\partial {H}_{z}}{\partial \rho }+\frac{1}{{\rho }^{2}}\frac{{\partial }^{2}{H}_{z}}{\partial {\phi }^{2}}+\frac{{\partial }^{2}{H}_{z}}{\partial {z}^{2}}+\frac{\varepsilon {\omega }^{2}}{{c}^{2}}{H}_{z}=0$$

The simplest solution is ${H}_{z}(z)=F(\rho ){e}^{im\phi }{e}^{i\beta z}$ and *F*(*ρ*) satisfies:

$$\frac{{\partial }^{2}F}{\partial {\rho }^{2}}+\frac{1}{\rho }\frac{\partial F}{\partial \rho }+(\frac{\varepsilon {\omega }^{2}}{{c}^{2}}-\frac{{m}^{2}}{{\rho }^{2}}-{\beta }^{2})F=0$$

*F* therefore satisfies Bessel equation in the core and modified Bessel equation outside the core. The simplest mode (*m* = 0) allows for TE characteristics. *H*_z_ is therefore:

$$\{\begin{array}{rcl}{H}_{z} & = & A{J}_{0}({k}_{\rho c}\rho ){e}^{i\beta z}\,{\rm{for}}\,\rho \le R\\ {H}_{z} & = & B{K}_{0}({k}_{\rho d}\rho ){e}^{i\beta z}\,{\rm{for}}\,\rho  > R\end{array},$$

and

$$\{\begin{array}{rcl}{{k}_{\rho c}}^{2} & = & \frac{{\omega }^{2}{\varepsilon }_{core}}{{c}^{2}}-{\beta }^{2}\\ {{k}_{\rho d}}^{2} & = & {\beta }^{2}-\,\frac{{\omega }^{2}{\varepsilon }_{dielectric}}{{c}^{2}}\end{array}$$

Here ${\varepsilon }_{core}$ is the permittivity of the cylindrical core and ${\varepsilon }_{d}$ the permittivity of the outside dielectric, with ${\varepsilon }_{core} > {\varepsilon }_{d}$. The solution for *E*_*φ*_ is then:

$$\{\begin{array}{ccc}{E}_{\phi } & = & \frac{i}{{{k}_{\rho c}}^{2}}(-{\mu }_{0}\omega \frac{{\rm{\partial }}{H}_{z}}{{\rm{\partial }}\rho })=\frac{iA}{{k}_{\rho c}}{\mu }_{0}\omega {J}_{1}({k}_{\rho c}\rho ){e}^{i\beta z}\,{\rm{f}}{\rm{o}}{\rm{r}}\,\rho \le R\\ {E}_{\phi } & = & \frac{i}{{{k}_{\rho d}}^{2}}({\mu }_{0}\omega \frac{{\rm{\partial }}{H}_{z}}{{\rm{\partial }}\rho })=\frac{-iB}{{k}_{\rho d}}{\mu }_{0}\omega {K}_{1}({k}_{\rho d}\rho ){e}^{i\beta z}\,{\rm{f}}{\rm{o}}{\rm{r}}\,\rho  > R\end{array}$$

And for *H*_*r*_ is:

$$\{\begin{array}{ccc}{H}_{r} & = & \frac{i}{{{k}_{\rho c}}^{2}}(\beta \frac{{\rm{\partial }}{H}_{z}}{{\rm{\partial }}\rho })=-\frac{iA\beta }{{k}_{\rho c}}{J}_{1}({k}_{\rho c}\rho ){e}^{i\beta z}\,{\rm{f}}{\rm{o}}{\rm{r}}\,\rho \le R\\ {H}_{r} & = & \frac{-i}{{{k}_{\rho d}}^{2}}(\beta \frac{{\rm{\partial }}{H}_{z}}{{\rm{\partial }}\rho })=\frac{iB\beta }{{k}_{\rho d}}{K}_{1}({k}_{\rho d}\rho ){e}^{i\beta z}\,{\rm{f}}{\rm{o}}{\rm{r}}\,\rho  > R\end{array}$$

The boundary conditions require *H*_*z*_ and *E*_*φ*_ continuous at *ρ* = *R*:

M1 $$B{K}_{0}({k}_{\rho d}R)=A{J}_{0}({k}_{\rho c}R)$$

M2 $$\frac{-B}{{k}_{\rho d}}{K}_{1}({k}_{\rho d}R)=\frac{A}{{k}_{\rho c}}{J}_{1}({k}_{\rho c}R)$$

The above solution is accurate for infinitely long cylindrically shaped waveguide.

### TE_01_ photonic mode in the dielectric-semiconductor (truncated cylindrical disk)-dielectric cavity and dielectric-semiconductor (truncated cylindrical disk)-dielectric-metal cavity

#### Semiconductor disk in dielectric

For a truncated semiconductor disk with top and bottom surface at z = ±a, the above solution is no longer valid. Assuming the dielectric material outside the semiconductor disk is the same everywhere, a good approximation for the electric and magnetic fields in the range of $|z|\le a$ is^5^:

$$\{\begin{array}{rcl}{H}_{z} & = & A{J}_{0}({k}_{\rho c}\rho )cos\beta z\,{\rm{for}}\,\rho \le R,|z|\le a\,\\ {H}_{z} & = & B{K}_{0}({k}_{\rho d}\rho )cos\beta z\,{\rm{for}}\,\rho  > R,|z|\le a\end{array}$$

$$\{\begin{array}{rcl}{E}_{\phi } & = & \frac{i}{{{k}_{\rho c}}^{2}}(-{\mu }_{0}\omega \frac{\partial {H}_{z}}{\partial \rho })=\frac{iA}{{k}_{\rho c}}{\mu }_{0}\omega {J}_{1}({k}_{\rho c}\rho )cos\beta z\,{\rm{for}}\,\rho \le R,|z|\le a\\ {E}_{\phi } & = & \frac{i}{{{k}_{\rho d}}^{2}}({\mu }_{0}\omega \frac{\partial {H}_{z}}{\partial \rho })=\frac{-iB}{{k}_{\rho d}}{\mu }_{0}\omega {K}_{1}({k}_{\rho d}\rho )cos\beta z\,{\rm{for}}\,\rho  > R,|z|\le a\end{array}$$

$$\{\begin{array}{rcl}{H}_{r} & = & \frac{1}{{{k}_{\rho c}}^{2}}(\frac{{\partial }^{2}{H}_{z}}{\partial z\partial \rho })=\frac{A\beta }{{k}_{\rho c}}{J}_{1}({k}_{\rho c}\rho )sin\beta z\,{\rm{for}}\,\rho \le R,|z|\le a\\ {H}_{r} & = & \frac{-1}{{{k}_{\rho d}}^{2}}(\frac{{\partial }^{2}{H}_{z}}{\partial z\partial \rho })=\frac{-B\beta }{{k}_{\rho d}}{K}_{1}({k}_{\rho }\rho )sin\beta z\,{\rm{for}}\,\rho  > R,|z|\le a\end{array}$$

Here,

M3 $${k}_{\rho c}^{2}=\frac{{\omega }^{2}{\varepsilon }_{core}}{{c}^{2}}-{\beta }^{2}$$

M4 $${k}_{\rho d}^{2}={\beta }^{2}-\,\frac{{\omega }^{2}{\varepsilon }_{dielectric}}{{c}^{2}}$$

The standing wave approximation along z direction indicates the partial reflection of the waves at the top and bottom surfaces due to the discontinuity of permittivity of the semiconductor disk and surrounding dielectric. In $|z| > a$ region, the solution to the Helmholtz equation is the superposition of a series of exponential decay and traveling Bessel functions (Hankel functions)^27^. We propose to use exponential decay function in z direction to approximate the evanescent fields in $\rho \le R\,and\,|z| > a$ region, as shown below. The radiative terms to the far field are only considered when estimating the radiation loss of the cavity as will be discussed in more details in next session.

$$\{\begin{array}{l}\begin{array}{ll}{H}_{z}=C{J}_{0}({k}_{\rho c}\rho ){e}^{-{k}_{d}|z|} & \mathrm{for}\,\rho \le R,|z| > a\\ {E}_{\phi }=\frac{i}{{{k}_{\rho c}}^{2}}(-{\mu }_{0}\omega \frac{\partial {H}_{z}}{\partial \rho })=\frac{iC}{{k}_{\rho c}}{\mu }_{0}\omega {J}_{1}({k}_{\rho c}\rho ){e}^{-{k}_{d}|z|}\, & \mathrm{for}\,\rho \le R,|z| > a\\ {H}_{r}=\frac{1}{{{k}_{\rho c}}^{2}}(\frac{{\partial }^{2}{H}_{z}}{\partial z\partial \rho })={(-1)}^{n}\frac{C{k}_{d}}{{k}_{\rho c}}{J}_{1}({k}_{\rho c}\rho ){e}^{-{k}_{d}|z|} & \mathrm{for}\,\rho \le R,|z| > a,\end{array}\\ \,\,\,\,\,\,\,n=0\,for\,z > 0\,and\,n=1\,for\,z < 0\end{array}$$

*k*_*d*_ satisfies:

M5 $$\,{k}_{\rho c}^{2}=\frac{{\omega }^{2}{\varepsilon }_{dielectric}}{{c}^{2}}+{k}_{d}^{2}$$

The relationship between constant *A* and *C* can be obtained by requiring *H*_r_ and *E*_φ_ continuous at *z* = ±*a*:

M6 $$A\beta sin\beta a=C{k}_{d}{e}^{-{k}_{d}a}$$

M7 $$Acos\beta a=C{e}^{-{k}_{d}a}$$

#### Semiconductor disk near metal

For the semiconductor disk on Al_2_O_3_/Ag or Al_2_O_3_/Cu, we can take a similar approach as we use to obtain the solution to the planar structure. We define an angle *θ* to describe how much the mode is pushed away from the middle of the semiconductor disk in z direction. The electric field can then be approximated by:

$$\{\begin{array}{rcl}{E}_{\phi } & = & \frac{i{\mu }_{0}\omega }{{k}_{\rho c}}{J}_{1}({k}_{\rho c}\rho ){B}_{t}{e}^{-{k}_{d}z}\,{\rm{for}}\,\rho \le R,z > a\\ {E}_{\phi } & = & \frac{i{\mu }_{0}\omega }{{k}_{\rho c}}{J}_{1}({k}_{\rho c}\rho )Acos(\beta z-\theta )\,{\rm{for}}\,\rho \le R,|z|\le a\\ {E}_{\phi } & = & \frac{i{\mu }_{0}\omega }{{k}_{\rho c}}{J}_{1}({k}_{\rho c}\rho )({B}_{b1}{e}^{{k}_{d}(z+a)}+{B}_{b2}{e}^{-{k}_{d}(z+a)})\,{\rm{for}}\,\rho \le R,\\  &  & \,-(a+h) < z < -\,a\\ {E}_{\phi } & = & \frac{i{\mu }_{0}\omega }{{k}_{\rho c}}{J}_{1}({k}_{\rho c}\rho )C{e}^{{k}_{m}(z+a+h)}\,{\rm{for}}\,\rho \le R,z < -\,(a+h)\end{array}$$

For the semiconductor disks near metal, we have:

M8 $${k}_{\rho c}^{2}=\frac{{\omega }^{2}{\varepsilon }_{core}}{{c}^{2}}-{\beta }^{2}$$

M9 $${k}_{\rho c}^{2}=\frac{{\omega }^{2}{\varepsilon }_{dielectric}}{{c}^{2}}+{k}_{d}^{2}$$

M10 $${k}_{\rho c}^{2}=\frac{{\omega }^{2}{\varepsilon }_{metal}}{{c}^{2}}+{k}_{m}^{2}$$

### Estimation of the resonant wavelength and quality factor of TE_01_ mode on glass and near metal

#### Estimation of TE_01_ resonant wavelength vs. disk radius

From Eq. (M1–M2), we can obtain:

M11 $$\frac{{k}_{\rho d}{K}_{0}({k}_{\rho d}R)}{{K}_{1}({k}_{\rho d}R)}+\frac{{k}_{\rho c}{J}_{0}({k}_{\rho c}R)}{{J}_{1}({k}_{\rho c}R)}=0$$

Eq. (M11) is valid for semiconductor disk in dielectric or near metal.

Combing Eq. (M3–M7), we have:

$$\{\begin{array}{rcl}\omega  & = & c\beta \sqrt{\frac{ta{n}^{2}\beta a+1}{{\varepsilon }_{core}-{\varepsilon }_{dielectric}}}\\ {k}_{\rho d} & = & \beta \sqrt{\frac{{\varepsilon }_{core}-2{\varepsilon }_{dielectric}-ta{n}^{2}\beta a{\varepsilon }_{dielectric}}{{\varepsilon }_{core}-{\varepsilon }_{dielectric}}}\\ {k}_{\rho c} & = & \beta \sqrt{\frac{{\varepsilon }_{dielectric}+ta{n}^{2}\beta a{\varepsilon }_{core}}{{\varepsilon }_{core}-{\varepsilon }_{dielectric}}}\end{array}$$

Plug above three equations into Eq. (M11), we can solve for the TE_01_ resonant frequency *ω* as a function of semiconductor disk radius *R* in a dielectric medium via the Newton-Raphson method. Best fit to COMSOL simulations with realistic material parameters leads to ${{\rm{\varepsilon }}}_{{\rm{core}}}=12.5$ and ${{\rm{\varepsilon }}}_{{\rm{dielectric}}}=2.5$ in the wavelength range of 500 nm to 1.3 µm, as shown in Fig. 3c.

Combining Eq. (M8–M10), we can obtain a similar dispersion relationship as Eq. (7) in the main texts for the semiconductor disk near metal:

M12 $$\frac{2\beta +tan(\beta a){k}_{d}-ctan(\beta a){k}_{d}}{2{k}_{d}-tan(\beta a)\beta +ctan(\beta a)\beta }=\,\frac{{k}_{d}+{k}_{m}-({k}_{d}-{k}_{m}){e}^{-2{k}_{d}h}}{{k}_{d}+{k}_{m}+({k}_{d}-{k}_{m}){e}^{-2{k}_{d}h}}$$

Combining Eq. (M11) and Eq. (M12), we obtain the TE_01_ resonant frequency *ω* as a function of semiconductor disk radius *R* for the disk near metal. Please note that in the analytical model we use dielectric-AlGaInP-dielectric-Ag structure in order to simplify the calculations. Best fit to COMSOL simulations with realistic material parameters leads to ${{\rm{\varepsilon }}}_{{\rm{core}}}=12.2$ and ${{\rm{\varepsilon }}}_{{\rm{dielectric}}}=2$ for the disks on Al_2_O_3_/Cu and ${{\rm{\varepsilon }}}_{{\rm{core}}}=12.5$ and ${{\rm{\varepsilon }}}_{{\rm{dielectric}}}=2$ for the disks on Al_2_O_3_/Ag in the wavelength range of 500 nm – 1 µm, as shown in Fig. 3c.

#### Estimation of TE_01_ Q vs. disk radius

To calculate the quality factor Q of the cavity, we need to know the radiation loss to the far field from the cavity and the internal loss of the cavity due to the absorption of the semiconductor material and the ohmic loss of the metal. As discussed in previous session, the accurate solution to Helmholtz equation in the far field is the superposition of a series of traveling Bessel functions and the calculation for radiation loss can be complicated^5^.

Here we propose a simplified model to estimate the radiation loss. We can calculate the equivalent polarization current density using the expression for polarization $P=({\varepsilon }_{c}-1){\varepsilon }_{0}E=\rho x.$ So, $\dot{P}=({\varepsilon }_{c}-1){\varepsilon }_{0}\dot{E}=\rho \dot{x}=J=$ $-\,i\omega ({\varepsilon }_{c}-1){\varepsilon }_{0}E$ in the semiconductor disk. Since TE_01_ only has *E*_*φ*_ component and is constant in φ direction, total electric dipole moment is zero. Therefore, the lowest order radiation is the magnetic dipole radiation. We can calculate the total magnetic dipole due to polarization current density for the semiconductor disk in dielectric as:

$$\begin{array}{l}\overrightarrow{m}=\frac{1}{2}\int \overrightarrow{r}\times \overrightarrow{J}dV\\ \,\,\,=\,\frac{-i\omega ({\varepsilon }_{c}-1){\varepsilon }_{0}}{2}(\int {\rho }^{2}{E}_{\phi }d\rho d\phi dz)\overrightarrow{z}-\frac{-i\omega ({\varepsilon }_{c}-1){\varepsilon }_{0}}{2}(\int z\rho {E}_{\phi }d\rho d\phi dz)\overrightarrow{\rho }\\ \,\,\,=\,2\pi \frac{A{\omega }^{2}sin\beta a}{{c}^{2}\beta {k}_{\rho c}}({\varepsilon }_{c}-1)(\int {\rho }^{2}{J}_{1}({k}_{\rho c}\rho )d\rho )\overrightarrow{z}\\ \,\,\,=\,2\pi \frac{A{\omega }^{2}sin\beta a}{{c}^{2}\beta {{k}^{2}}_{\rho c}}({\varepsilon }_{c}-1){R}^{2}{J}_{2}({k}_{\rho c}R)\overrightarrow{z}\end{array}$$

For a magnetic dipole, its radiation power is ${P}_{rloss}=\frac{{\mu }_{0}{\omega }^{4}{|m|}^{2}}{12\pi {c}^{3}}$^19^. Plugging the expression of *m* into this equation, we get:

$$\begin{array}{rcl}{P}_{rloss} & = & \frac{{\mu }_{0}{\omega }^{4}}{12\pi {c}^{3}}{|2\pi \frac{A{\omega }^{2}sin\beta a}{{c}^{2}\beta {{k}^{2}}_{\rho c}}({\varepsilon }_{c}-1){R}^{2}{J}_{2}({k}_{\rho c}R)\overrightarrow{z}|}^{2}\\  & = & \frac{{\mu }_{0}{\omega }^{8}\pi {A}^{2}si{n}^{2}\beta a}{3{c}^{7}{\beta }^{2}{k}_{\rho c}^{4}}{({\varepsilon }_{c}-1)}^{2}{R}^{4}{J}_{2}{({k}_{\rho c}R)}^{2}\end{array}$$

${\varepsilon }_{c}$ is in general a complex number. In the estimation of the radiation loss, we only use the real part of ${\varepsilon }_{c}.$The internal loss ${P}_{iloss}$ is estimated for the entire volume using^19^:

$${P}_{iloss}=\iiint \frac{1}{2}{\varepsilon }_{0}Im(\varepsilon )\omega |E{|}^{2}d{\boldsymbol{V}}$$

The stored energy inside the semiconductor disk is calculated using^5^:

$${\rm{U}}=\iiint ud{\boldsymbol{V}}=\iiint \frac{1}{2}({\varepsilon }_{0}\frac{{\rm{\partial }}({\varepsilon }_{c}\omega )}{{\rm{\partial }}\omega }{E}^{2}+{\mu }_{0}{H}^{2})d{\boldsymbol{V}}$$

In the case of TE_01_ mode, the electric field only has ${E}_{\phi }$ component:

$$\begin{array}{ccc}\iiint {E}_{\phi }^{2}d{\boldsymbol{V}} & = & {\int }_{0}^{R}{\int }_{0}^{2\pi }{\int }_{-a}^{a}{E}_{\phi }^{2}\rho d\rho d\phi dz\\  & = & \frac{{A}^{2}{\mu }_{0}^{2}{\omega }^{2}\pi {R}^{2}}{{k}_{\rho c}^{2}}({\rm{a}}+\frac{sin2\beta a}{2\beta })[{J}_{1}^{2}({k}_{\rho c}R)-{J}_{0}({k}_{\rho c}R){J}_{2}({k}_{\rho c}R)]\end{array}$$

$$\iiint {H}_{z}^{2}d{\boldsymbol{V}}={A}^{2}\pi {R}^{2}({\rm{a}}+\frac{sin2\beta a}{2\beta })[{J}_{0}^{2}({k}_{\rho c}R)+{J}_{1}^{2}({k}_{\rho c}R)]$$

$$\iiint {H}_{r}^{2}d{\boldsymbol{V}}=\frac{{A}^{2}{\beta }^{2}\pi {R}^{2}}{{k}_{\rho c}^{2}}({\rm{a}}-\frac{sin2\beta a}{2\beta })[{J}_{1}^{2}({k}_{\rho c}R)-{J}_{0}({k}_{\rho c}R){J}_{2}({k}_{\rho c}R)]$$

The quality factor Q can then be calculated using

$$Q=\frac{U}{{P}_{rloss}+{P}_{iloss}}$$

Similar calculations can be carried out on the semiconductor disk on Al_2_O_3_/Ag and on Al_2_O_3_/Cu. Using the electric field expressions detailed in previous session, we have:

$$\begin{array}{rcl}\overrightarrow{m} & = & 2\pi \frac{A{\omega }^{2}\,\sin \,\beta acos\theta }{{c}^{2}\beta {k}_{\rho c}^{2}}({\varepsilon }_{c}-1){R}^{2}{J}_{2}({k}_{\rho c}R)\overrightarrow{z}\\  &  & +\,\pi \frac{{\omega }^{2}({B}_{b1}(1-{e}^{-{k}_{d}h})+{B}_{b2}(-1+{e}^{{k}_{d}h}))}{{c}^{2}{k}_{\rho c}^{2}{k}_{d}}({\varepsilon }_{d}-1){R}^{2}{J}_{2}({k}_{\rho c}R)\overrightarrow{z}\\  &  & +\,\pi \frac{{\omega }^{2}C}{{c}^{2}{k}_{\rho c}^{2}{k}_{m}}({\varepsilon }_{m}-1){R}^{2}{J}_{2}({k}_{\rho c}R)\overrightarrow{z}\end{array}$$

It is clear from the above expression that the first term is similar to the magnetic dipole $\overrightarrow{m}$ of the semiconductor disk in a dielectric medium, apart from being multiplied by a factor of cos*θ* due to the presence of the metal substrate, which reduces the total magnetic dipole moment. The third term is the induced magnetic dipole by the image current of the metal in metal region. Since $({\varepsilon }_{m}-1)$ is negative, the third term is in opposite direction to the first term. As a result, the magnetic dipole $\overrightarrow{m}$ of the semiconductor disk on Al_2_O_3_/Ag and on Al_2_O_3_/Cu is substantially decreased compared to its counterpart in a dielectric medium and higher quality factor is achieved in the semiconductor disk on Al_2_O_3_/Ag and on Al_2_O_3_/Cu. The estimated Q of semiconductor disk in dielectric and near metal are plotted in Fig. 3(d). Please note that in the analytical model we use dielectric-AlGaInP-dielectric-Ag structure. The effective relative permittivity of the dielectric layer is obtained from the best fit to Fig. 3(c), in order to keep the analytical model self-consistent between Fig. 3(c) and Fig. 3(d). The loss of cavity is more sensitively related to the dielectric and insulator used in the COMSOL simulation than the resonant wavelength of the cavity. Nevertheless, we do think our analytical model reasonably reproduces the transition from radiation loss domination to internal loss domination region of the Q factors.

## Electronic supplementary material

Supplementary information

## Data Availability

The datasets generated during and/or analysed during the current study are available from the corresponding author on reasonable request.

## References

[1] Smit M, van der Tol J, Hill M (2012). Moore’s law in photonics. Laser & Photonics Reviews.

[2] Ding K, Ning CZ (2013). Fabrication challenges of electrical injection metallic cavity semiconductor nanolasers. Semicond. Sci. Technol..

[3] Hill MT (2007). Lasing in metallic-coated nanocavities. Nature Photonics.

[4] Nezhad MP (2010). Room-temperature subwavelength metallo-dielectric lasers. Nature Photonics.

[5] Manolatou C, Farhan R (2008). Subwavelength Nanopatch Cavities for Semiconductor Plasmon Lasers. Ieee Journal of Quantum Electronics.

[6] Yu K, Lakhani A, Wu MC (2010). Subwavelength metal-optic semiconductor nanopatch lasers. Optics Express.

[7] Hill MT, Gather MC (2014). Advances in small lasers. Nature Photonics.

[8] Oulton RF (2009). Plasmon lasers at deep subwavelength scale. Nature.

[9] Zhang Q (2014). A room temperature low-threshold ultraviolet plasmonic nanolaser. Nature Communications.

[10] Lu YJ (2012). Plasmonic nanolaser using epitaxially grown silver film. Science.

[11] Lu Y-J (2014). All-Color Plasmonic Nanolasers with Ultralow Thresholds:Autotuning Mechanism for Single-Mode Lasing. Nano Letters.

[12] Ma R-m, Oulton RF, Sorger VJ, Bartal G, Zhang X (2011). Room-temperature sub-diffraction-limited plasmon laser by total internal reflection. Nature Materials.

[13] Liu N (2016). Lithographically Defined, Room Temperature Low Threshold Subwavelength Red-Emitting Hybrid Plasmonic Lasers. Nano Letters.

[14] Lakhani AM, Kim M-k, Lau EK, Wu MC (2011). Plasmonic crystal defect nanolaser. Optics Express.

[15] Chou YH (2016). High-Operation-Temperature Plasmonic Nanolasers on Single-Crystalline Aluminum. Nano Letters.

[16] Costantini D (2013). A hybrid plasmonic semiconductor laser. Appl. Phys. Lett..

[17] Maier, S. A. *Plasmonics: Fundamentals and Applications*. (Springer, 2007).

[18] Schubert M (1995). Optical constants of GaxIn1-xP lattice matched to GaAs. J. Appl. Phys..

[19] Jackson, J. D. *Classical Electrodynamics*. 3rd edn, (John Wiley & Sons, Inc, 1999).

[20] Cohn SB (1968). Microwave bandpass filters containing high-Q dielectric resonators. IEEE Trans. Microw. Theory Tech..

[21] Elisabet Xifre-Perez (2013). Mirror-Image-Induced Magnetic Modes. ACS nano.

[22] Huang Z (2015). Strong-Field-Enhanced Spectroscopy in Silicon Nanoparticle Electric and Magnetic Dipole Resonance near a Metal Surface. J. Phys. Chem. C.

[23] Rakic AD, Djurisic AB, Elazar JM, Majewski ML (1998). Optical properties of metallic films for vertical-cavity optoelectronic devices. Appl. Opt..

[24] Huang Z (2014). Nanoscale active hybrid plasmonic laser with a metal-clad metal–insulator–semiconductor square resonator. J. Opt. Soc. Am. B.

[25] Kwon S-H (2012). Deep subwavelength plasmonic whisperinggallery-mode cavity. Optics Express.

[26] Snyder, A. W. & Love, J. D. *Optical Waveguide Theory*. (Chapman and Hall, 1983).

[27] Balanis, C. A. *Antenna Theory*. 2nd edn, (John Wiley & Sons, Inc, 1997).

