# Supplementary material for: Low loss photonic nanocavity via dark magnetic dipole resonant mode near metal
Source: Sci Rep. 2018 Nov 19;8:17054. doi: 10.1038/s41598-018-35291-w (PMC6242897; doi:10.1038/s41598-018-35291-w)
Supplement: Supplementary file 1 — Supplementary information [file 41598_2018_35291_MOESM1_ESM.docx]

**Low loss photonic nanocavity via dark magnetic dipole resonant mode near metal**

Ning Liu^*1^, Christophe Silien^1^, Greg Sun^2^, Brian Corbett^3^

^1^*Department of Physics and Bernal Institute, University of Limerick, Limerick, Ireland*

*^2^Department of Engineering, University of Massachusetts Boston, Boston, MA 02125*

^3^*Tyndall National Institute, University College Cork, Cork, Ireland*

**Supporting Information**

1. ***H_z_ solution of TE mode in the dielectric-semiconductor-dielectric-metal 4-layered planar structure***

To complete the solution of the simplest TE mode in the 4 layered structure of planar geometry as given in the main texts, the H_z_ components are given below:

$$\left\{ \begin{matrix} \begin{matrix} H_{z}\left( z \right)=B_{t}{\frac{\beta}{\omega\mu_{0}}e}^{i\beta x}e^{{-k}_{d}(z-a)} \\ H_{z}\left( z \right)=A{\frac{\beta}{\omega\mu_{0}}e}^{i\beta x}\cos\left( k_{c}z-\theta\right) \\ H_{z}\left( z \right)=B_{b1}{\frac{\beta}{\omega\mu_{0}}e}^{i\beta x}e^{k_{d}\left( z+a \right)}+B_{b2}{\frac{\beta}{\omega\mu_{0}}e}^{i\beta x}e^{{-k}_{d}(z+a)} \end{matrix} \\ H_{z}\left( z \right)=C{\frac{\beta}{\omega\mu_{0}}e}^{i\beta x}e^{k_{m}(z+a+h)} \end{matrix} \right. \begin{matrix} \mathrm{for} z>a \\ \mathrm{for} |z|<a \\ \mathrm{for} -\left( a+h \right)<z<-a \\ \mathrm{for} z<-(a+h) \end{matrix}$$

1. ***Plasmonic mode at a single dielectric-metal interface***

Starting from the central equation of electromagnetic wave theory:

$\nabla^{2}\mathbf{E-}\frac{\boldsymbol{\varepsilon}}{\boldsymbol{c}^{\boldsymbol{2}}}\frac{\boldsymbol{\partial}^{\boldsymbol{2}}\mathbf{E}}{\boldsymbol{\partial}\boldsymbol{t}^{\boldsymbol{2}}}\boldsymbol{=}0$


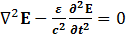


Assuming the **E** has a single harmonic time dependence **E**(**r**,t)=**E**(**r**)e*^-iωt^*, we can then obtain Helmholtz equation:

$\nabla^{2}\mathbf{E+}\frac{\omega^{2}\varepsilon}{c^{2}}\mathbf{E}\boldsymbol{=}0$ (**S1**)

For a single dielectric-metal interface, there are only two media and one interface. We choose x as the propagation direction of the plasmonic wave and z the direction perpendicular to the infinitely large planes. The solution to Eq. (S1) can be found from ref. [[1](#_ENREF_17)]. The boundary conditions allow only TM mode to propagate at the interface of infinitely large planes, with ${k_{d}}/{k_{m}=-{\varepsilon_{d}}/{\varepsilon_{m}}}$, and $k_{i}^{2}=\beta^{2}-\frac{\omega^{2}\varepsilon_{i}}{c^{2}}$ (*i=d,m* and *k_i_* > 0). Combining these three equations, we can obtain the well known surface plasmon dispersion relation at a single interface:


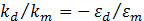


$\omega=\beta c\sqrt{\frac{\varepsilon_{m}+\varepsilon_{d}}{\varepsilon_{m}\varepsilon_{d}}}$ (**S2**)


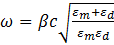


The analytical solution to Eq. (S2), assuming $\varepsilon_{m}=1-\frac{\omega_{p}^{2}}{\omega^{2}}$ is:

$$\omega^{2}=\frac{\omega_{p}^{2}}{2}\left\{ 1+\frac{\beta^{2}c^{2}}{{\varepsilon_{d}\omega}_{p}^{2}}\left( 1+\varepsilon_{d} \right)-\sqrt{1+\frac{\beta^{4}c^{4}}{{{\varepsilon_{d}}^{2}\omega}_{p}^{4}}\left( 1+\varepsilon_{d} \right)^{2}+\frac{{2\beta}^{2}c^{2}}{{\varepsilon_{d}\omega}_{p}^{2}}\left( \varepsilon_{d}-1 \right)} \right\}$$

The above formula is used to calculate the plasmonic dispersion curves at a single metal-dielectric interface used in Fig. 1c. For real transition metals (Ag, Au, Cu) with *ω* < *ω_p_*, the permittivity is better described by $\varepsilon_{m}=\varepsilon_{\infty}-\frac{\omega_{p}^{2}}{\omega^{2}+i\gamma\omega}$ [1], where γ is a damping constant. Simulation results presented in Fig. 1d, Fig. 2 and Fig. 3 are obtained using realistic material parameters.

1. ***Guided fundamental TE mode in the dielectric-semiconductor-dielectric planar structure***

For a multilayer (> 2 layers) system, we can solve Eq. (S1) in each domain and determine their coefficients using appropriate boundary conditions. For the simplest 3 layered system, we can choose the top and bottom domains the same materials, with the core using a different material. In photonic case, both the core and cladding materials are dielectric with *ε* > 0. In this case, when *ε_core_ > ε_cladding_*, guided modes are supported. Both TE and TM modes can be achieved. If we choose z = 0 at the middle of the core layer and let thickness of the core be 2a. For the lowest order TE mode, the electric and magnetic fields satisfy [2,[3](#_ENREF_23)]:

$\left\{ \begin{matrix} E_{y}\left( z \right)=B_{t}e^{i\beta x}e^{{-k}_{d}(z-a)} \\ H_{x}\left( z \right)={-iB}_{t}{\frac{k_{d}}{\omega\mu_{0}}e}^{i\beta x}e^{-k_{d}(z-a)} \\ H_{z}\left( z \right)=B_{t}{\frac{\beta}{\omega\mu_{0}}e}^{i\beta x}e^{{-k}_{d}(z-a)} \end{matrix} \mathrm{for} z>a \right.$


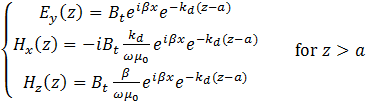


$\left\{ \begin{matrix} E_{y}\left( z \right)=Ae^{i\beta x}cos(k_{c}z) \\ H_{x}\left( z \right)=-iA{\frac{k_{c}}{\omega\mu_{0}}e}^{i\beta x}sin(k_{c}z) \\ H_{z}\left( z \right)=A{\frac{\beta}{\omega\mu_{0}}e}^{i\beta x}cos(k_{c}z) \end{matrix} \mathrm{for} |z|<a \right.$


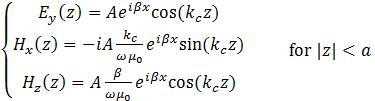

$$\left\{ \begin{matrix} E_{y}\left( z \right)=B_{b}e^{i\beta x}e^{k_{d}(z+a)} \\ H_{x}\left( z \right)={iB}_{b}{\frac{k_{d}}{\omega\mu_{0}}e}^{i\beta x}e^{k_{d}(z+a)} \\ H_{z}\left( z \right)=B_{b}{\frac{\beta}{\omega\mu_{0}}e}^{i\beta x}e^{k_{d}(z+a)} \end{matrix} \mathrm{for} z<-a \right.$$

Continuity of *E_y_* and *H_x_* at the interface leads to the conditions that $B_{b}=B_{t}=Acos(k_{c}a)$ and $\tan k_{c}a=\frac{k_{d}}{k_{c}}$, with $\left\{ \begin{matrix} k_{c}^{2}=\frac{\omega^{2}\varepsilon_{c}}{c^{2}}-\beta^{2} \\ k_{d}^{2}=\beta^{2}-\frac{\omega^{2}\varepsilon_{d}}{c^{2}} \end{matrix} \right.$ .


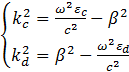


1. ***Electric field distribution of TE mode in photonic 3 layered planar structure, TE mode in 4 layered planar structure and TM mode in 4 layered planar structure***


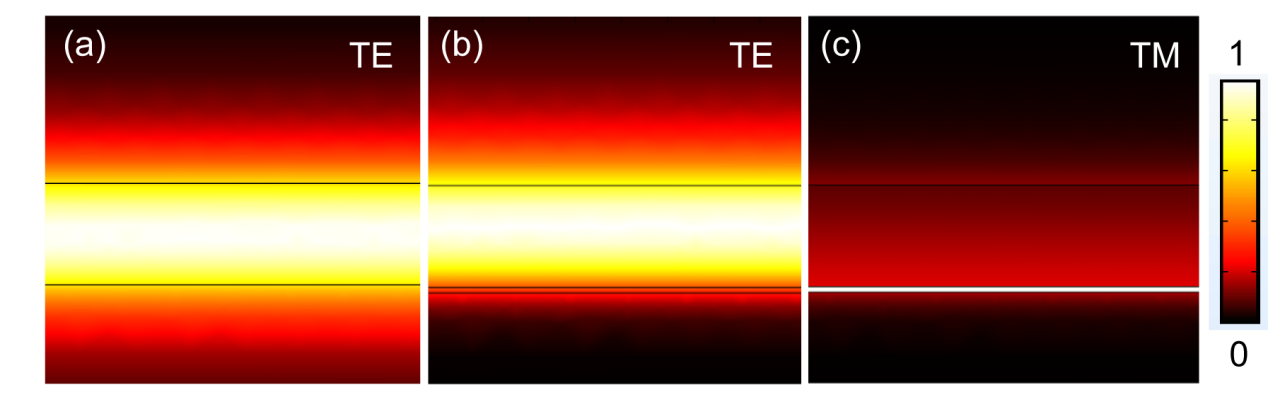


**Figure S1** (a-c) Normalized E field |E| distribution of TE mode in the dielectric-semiconductor-dielectric 3 layered structure, TE mode in the dielectric-semiconductor-dielectric-metal 4 layered structure and TM mode in the dielectric-semiconductor-dielectric-metal 4 layered structure, respectively. In this 2D simulation, d = 110 nm, h = 6 nm, ε_core_ = 13, ε_d_ = 2.9 and wavelength is 820 nm. The metal is assumed lossless with plasma frequency at 2.27×10^15^ Hz.

1. ***Additional field distribution of TE_01_ on glass and TE_01_ and TM_11_ modes on Al_2_O_3_/Ag***


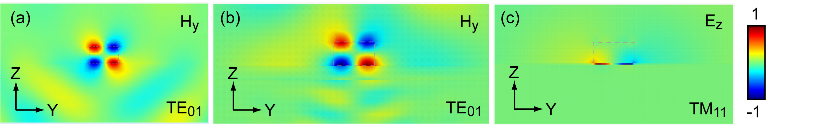


**Figure S2** (a, b) COMSOL simulations of normalized TE_01_ mode magnetic field H_y_ distribution on zy plane at x = 0, for semiconductor disk on glass and semiconductor disk on Al_2_O_3_/Ag (diameter of 200 nm for both cases). (c) Normalized TM_11_ mode electric field E_z_ distribution on zy plane at x = 0, for semiconductor disk (200 nm in diameter) on Al_2_O_3_/Ag. The dashed rectangles indicate the physical contours of the semiconductor disks.

1. ***Q of TE_01_ on Al_2_O_3_/Ag as a function of Al_2_O_3_ thickness***


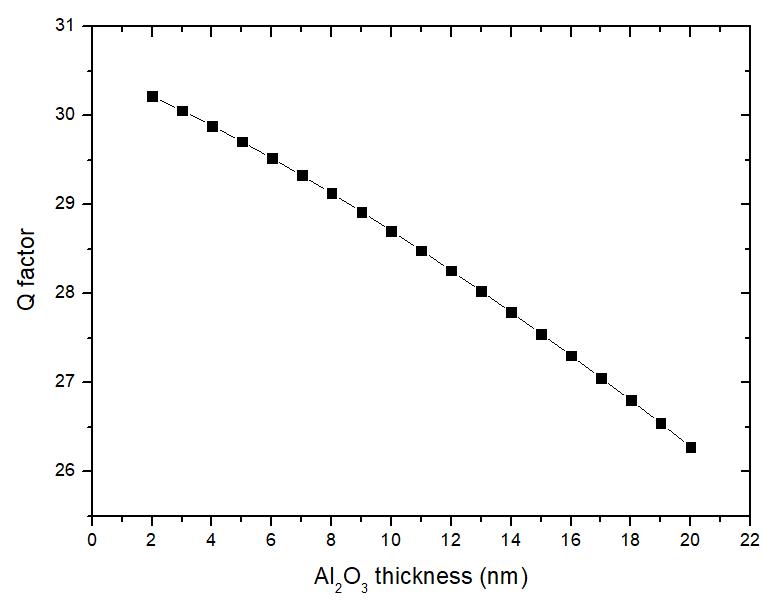


**Figure S3** COMSOL simulated quality factor Q of TE_01_ mode on Al_2_O_3_//Ag vs. the thickness of

Al_2_O_3_. In this simulation, the diameter of the AlGaInP disk is fixed at 200 nm.

Reference:

1. S. A. Maier, *Plasmonics: Fundamentals and Applications* (Springer, 2007).
2. J. D. Jackson, *Classical Electrodynamics* (John Wiley&Sons, Inc., New York, 1999), 3rd edn.
3. D. K. Cheng, *Field and wave electromagnetics* (Addison – Wesley Publishing Company, Inc., 1983).
